# First Evidence for Adoption in California Sea Lions

**DOI:** 10.1371/journal.pone.0013873

**Published:** 2010-11-08

**Authors:** Ramona Flatz, Leah R. Gerber

**Affiliations:** School of Life Sciences, Arizona State University, Tempe, Arizona, United States of America; University of Plymouth, United Kingdom

## Abstract

Demographic parameters such as birth and death rates determine the persistence of populations. Understanding the mechanisms that influence these rates is essential to developing effective management strategies. Alloparental behavior, or the care of non-filial young, has been documented in many species and has been shown to influence offspring survival. However, the role of alloparental behavior in maintaining population viability has not been previously studied. Here, we provide the first evidence for adoption in California sea lions and show that adoption potentially works to maintain a high survival rate of young and may ultimately contribute to population persistence. Alloparental behavior should have a positive effect on the population growth rate when the sum of the effects on fitness for the alloparent and beneficiary is positive.

## Introduction

Alloparental behavior, the care of non-filial young, has been widely documented in mammal and bird species [1]–[3]. While the benefits to young (e.g. increased survival) are apparent [3] and understanding the mechanisms that determine demographic rates (i.e. survival and reproduction) is essential for effective conservation and management [4]–[6], the influence of alloparental care on demographic rates has remained largely overlooked in the ecology and conservation literature.

In California sea lions (*Zalophus californianus*), females are able to recognize and discriminate non-filial young, and pups show strong preference for their mother [7], [8]. Thus, incidents of non-filial nursing in this species are expected to be rare [7]–[9] and likely represent adoption of orphaned pups by females who have miscarried or lost pups [1], [2]. Because pups depend on their mother for survival during their first year [7], [8], orphaned pups would die if not adopted by lactating females. Thus, these adoption events have the potential to reduce pup mortality rates, contributing to population persistence. In this paper, we provide the first evidence for adoption in California sea lions. We then examine the role of alloparental behavior in maintaining survival of young and discuss consequences for population viability.

## Methods

### Ethics statement

All procedures were approved by the Arizona State University Animal Care and Use Committee (07-918R).

### Sample collection

**Figure 1:**
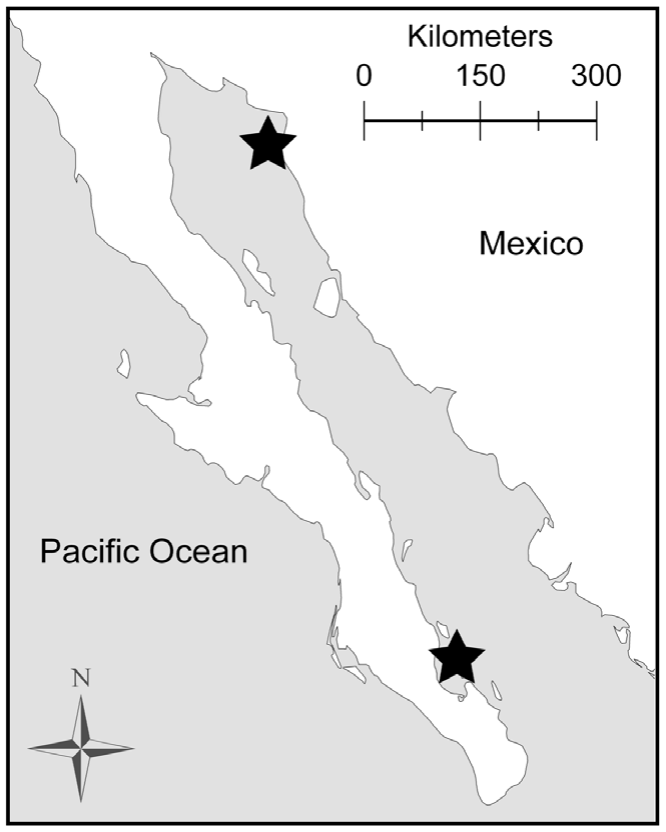
Map of the study sites. Study sites included breeding colonies on San Jorge Island (north) and Los Islotes Island (south), in the Gulf of California, Mexico.

Pups were captured at approximately 4 days to 8 weeks of age in June and July of 2005–2008 at San Jorge and Los Islotes Islands in the Gulf of California (Figure 1). During capture sessions morphological measurements were taken, pups were marked with unique haircuts, and toe clips were taken for genetic analysis. Additionally, pups captured in July were given flipper tags for long-term identification. Female biopsies were taken using a crossbow and bolts fitted with biopsy tips (Quality Manufacturing, Inc.) attached to a fishing line [10]. To ensure sampling of female-pup pairs, biopsies were obtained from females only when they were nursing a marked pup. All biopsies were handled with sterilized tweezers and stored in 2.0 µl vials containing 90% ethanol.

### Genetic analyses

DNA was isolated from tissue samples and amplified at 14 microsatellite loci (Table S1) using the QIAGEN multiplex PCR kit (QIAGEN, Inc.). Fragment analysis was conducted on an ABI 3730 DNA Analyzer (Applied Biosystems, Inc.) and loci were scored by hand using the program GeneMapper v4.0. The program CERVUS [11] was used to identify mismatched female-pup genotypes. Mismatches between female and pup genotypes represent loci for which neither of the two possible alleles present in the female genotype were present in the pup genotype. Repeated genotyping of 10% of the samples showed that the genotyping error rate was low (0.0014%). Additionally, we repeated PCR and fragment analysis on mismatched female-pup pairs to minimize false conclusions due to genotyping error. Mismatches at only 1 locus (n = 2) were not considered as evidence of non-filial nursing because of possible genotyping error or mutation [12]. Errors in identifying samples during the extraction process would potentially result in female-pup mismatches at multiple loci. To address this, the genotypes of mismatched females were compared to the pool of pup genotypes and vice versa. There were no cases where mismatched females or pups matched any other individual at all loci.

Genetic relatedness between female-pup pairs was calculated with the Microsoft Excel Macro ‘GROUPRELATE’ [13]. Using this program, group relatedness is estimated by averaging values for pairwise relatedness [14] between all individuals in a group. Thus, to obtain a relatedness value (r-value) for each female-pup pair, we defined each group as consisting of one female-pup pair. Pairwise relatedness was calculated for both filial and non-filial female-pup pairs as determined by CERVUS results. Using the same individuals from filial and non-filial female-pup pairs, we analyzed relatedness between randomly assigned female-pup pairs; the resulting r-values served as a baseline with which to compare r-values from non-filial female pup pairs.

### Population viability analysis

To examine the potential consequences of adoption for population viability, we estimated the discrete rate of annual population growth (λ) based on a Leslie matrix model with fecundity and survival estimates for 19 age classes at Los Islotes Island [6]. We assumed that adopted pups would otherwise not survive to the next age class and that there is no difference in lifetime survival and reproductive output between adopted and filial young. With the simplistic assumption of exponential population growth, we modeled three scenarios of adoption in the population. First, we assumed that the current vital rates (i.e. no change in pup survival or female fecundity) represent a scenario where adoption occurs at no cost to the alloparent. Second, we considered a scenario where adoption represents a cost to the alloparent. We assumed that an adopting female in year t would not reproduce in year t+1, which leads to a reduction in fecundity. We first calculated the adopting rate of females by dividing the fraction of the population adopted among the females in age classes 5–19 (sexually mature females), we then reduced fecundity in age classes >5 by the adopting rate. Third, we modeled population growth in the absence of these adoption events by reducing 1^st^ year survival by the adoption rate. We considered a range of adoption rates with a maximum adoption rate of 15% based on our genotyping results. To illustrate the effects of small changes in λ on long-term abundance, we solved for the equation N_t_ = N_0_ λ^t^ where t = 50 and N_0_ = 439 (representing the most recent estimate of abundance for Los Islotes Island [15]).

## Results

### Non-filial nursing in California sea lions

**Figure 2:**
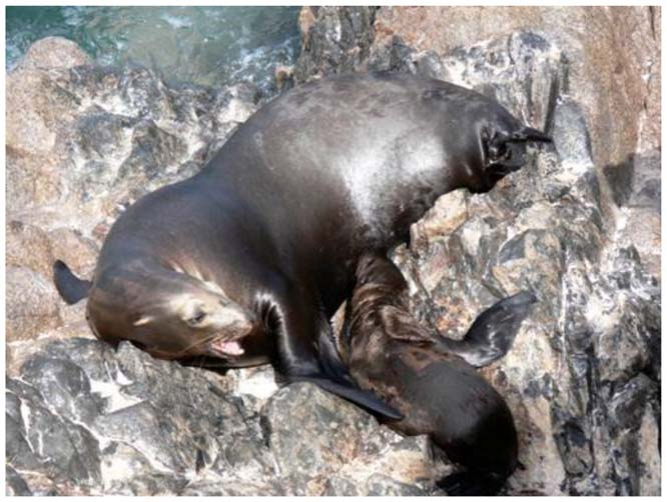
An adult female and her adopted pup. Female nursing a non-filial pup marked with the haircut ‘A1’. Unique scars on this female, particularly the absence of both hind flippers, allowed researchers to identify her and her pup throughout the field season.

We documented mismatches at ≥2 loci for 6 out of 109 sampled female-pup pairs from San Jorge Island, and 9 out of 51 pairs from Los Islotes Island. Additionally, we documented adoption events for two female-pup pairs at San Jorge Island. In both cases, females exhibited distinctive scar patterns, allowing us to track both the female and pup over time. In the first case, the female-pup pair was first identified (and sampled) in August 2007. In October of 2008, the same female was observed nursing the pup tagged in 2007 while simultaneously nursing a new pup from 2008. In the second case, a marked female was observed calling for her pup and receiving no response for three days in June 2008. In July and August of the same year, she was observed nursing a marked pup on multiple occasions (Figure 2). We subsequently obtained a biopsy from this female based on our suspicions that she may have adopted the pup. This represents the only instance of non-random sampling, and was not included in the six cases of non-filial nursing from San Jorge Island or used in subsequent analyses. For each case we found mismatches between the mother and pup genotypes at 5 and 3 of the 14 loci, respectively. Our observations represent the first documented cases of adoption in California sea lions.

**Figure 3:**
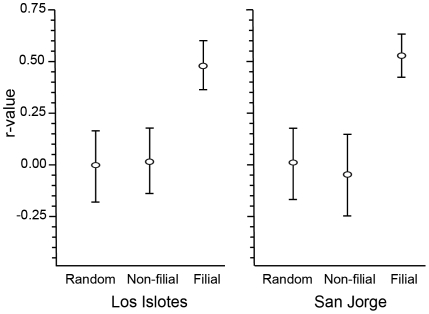
Genetic relatedness between female-pup pairs. Mean pairwise relatedness (r-values) between filial, non-filial, and randomly generated female-pup pairs on Los Islotes and San Jorge rookeries. Relatedness between non-filial female-pup pairs is no different than expected at random. Error bars represent 1 standard deviation from the mean.

Because there are significant differences in background allele frequencies between San Jorge and Los Islotes Islands [16], we calculated r-values for female-pup pairs separately at each island. All identified non-filial female-pup pairs and 80 filial female-pup pairs (40 from each island) were used in relatedness analysis. R-values between filial female-pup pairs were significantly higher than zero with a mean r≈0.5, as is expected in first order relatives [14]. Mean r-values did not significantly differ from zero in both the randomly assigned and the non-filial female-pup pairs (Figure 3).

### Implications for estimates of population growth rate

**Figure 4:**
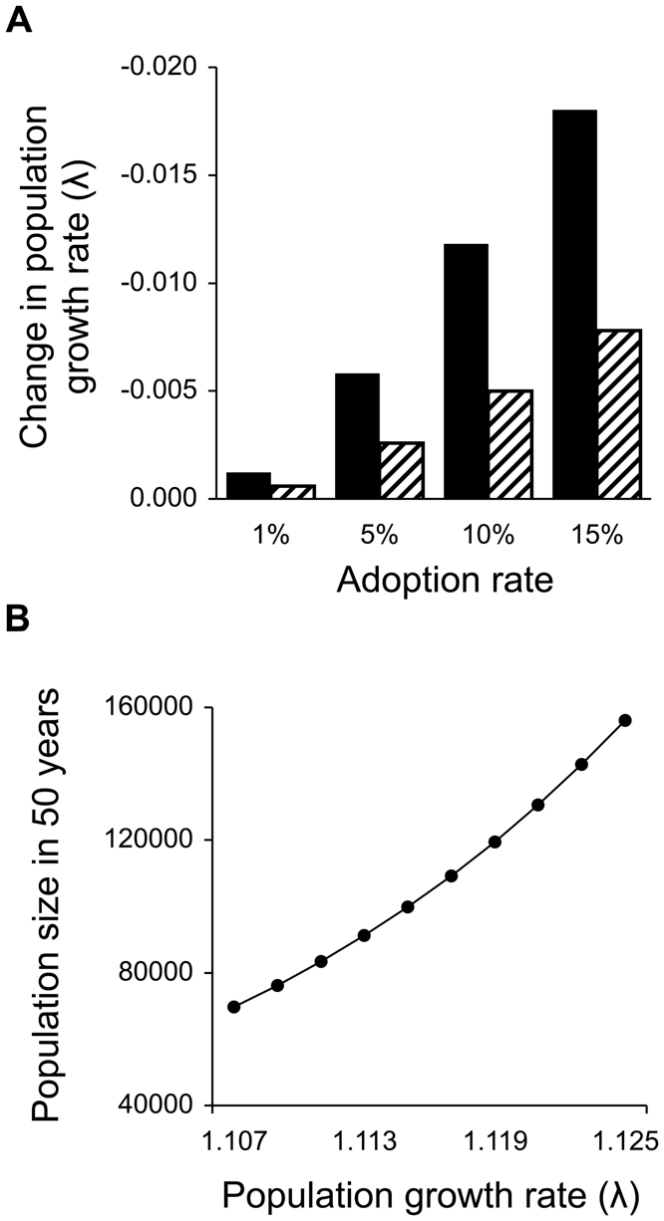
The potential impact of adoption on population growth. Increased pup survival rates result from adoption of orphaned pups and potentially affect the overall population growth rate and long-term population viability. a) Change in population growth rate (λ) resulting from reductions in pup survival in the absence of adoption (solid bars) and reductions in female fecundity as a cost to adopting (hashed bars) for a range of observed adoption rates; b) Projected abundance at Los Islotes in 50 years for a range of underlying rates of population growth under alternative assumptions about adoption reflecting the small changes in λ portrayed in Figure 4a.

We estimated λ = 1.125 for Los Islotes Island based on vital rates reported in Gerber [6]. To examine the potential role of adoption in the context of population viability, we modeled three scenarios of adoption in the population. First, we assumed that the current vital rates reflect adoption occurring at no cost to the alloparent (λ = 1.125). Second, when adoption was modeled at a cost to the alloparent, pup survival remained constant and female fecundity was reduced by 0.5%–7.2% for adoption rates of 1%–15%, respectively. This resulted in λ = 1.117–1.124 (Figure 4a), demonstrating that, even with a fitness cost, adoption can have a positive effect on population growth. Third, to consider population growth in the absence of these adoption events by reducing 1^st^ year survival by the adoption rate, we found λ = 1.107–1.124 for adoption rates of 1%–15% (Figure 4a). Even these small reductions in λ have the potential to decrease long-term population size (Figure 4b).

## Discussion

### Adoption in California sea lions

In this study, we found that adoption occurs in natural populations of California sea lions by combining genotypic and behavioral data from two female-pup pairs. Repeated observations of these individuals confirmed that, although non-filial, each relationship mirrored that of conventional female-pup pairs. A more extensive analysis of genetic maternity suggested that adoptive female-pup pairs accounted for less than 6% of the female-pup pairs on San Jorge Island. This is consistent with studies of other otariids (fur seals and sea lions), including the Steller sea lion [17], New Zealand sea lion [18], and Antarctic fur seal [12]. Surprisingly, over 17% of the female-pup pairs on Los Islotes Island were non-filial, which represents the highest rate of non-filial nursing ever reported for any otariid. The difference in the frequency of adoption between islands may have resulted from differences in disturbance, environment, and/or demography [1], [3], [19]. Further research is needed to determine which, if any, of these factors influence the rate of adoption in California sea lions.

Because most females do not bear identifying marks, incidents where a pup nursed from multiple females or where a female nursed two or more pups on separate occasions would have gone undetected. Thus, it is possible that mismatches do not always represent actual adoption events. Instead mismatches could result from pups stealing milk from unrelated females, or reciprocal nursing (i.e. females willingly nurse each other's pups) [3], [17]. This is often the case in phocids (true seals), which have poorly developed methods for mother-pup recognition resulting in high rates of non-filial nursing [2], [7], [9]. However, incidents of milk stealing are rare in otariids due to highly developed mechanisms for mother-pup recognition and an exclusive mother-pup bond [7]–[9], [17], [20], [21]. Furthermore, in otariids, female aggression toward milk-stealers makes such events brief and easily identifiable by researchers [7], [17], [21].

Reciprocal nursing is generally restricted to cases where nearly all females participate [3], [22] and is thus highly unlikely given the low rate of alloparenting in otariids [7], [8]. Reciprocity could be maintained at low frequencies if females selectively nurse offspring of close relatives and gain inclusive fitness benefits [3]. Thus, non-filial nursing via kin selection seems plausible among otariids given their ability of individual recognition. However, our analysis of relatedness between filial and non-filial female-pup pairs showed no evidence of kin selection. The similarity in relatedness estimates between non-filial and randomly assigned female-pup pairs also indicates that female-pup mismatches were not due to genotyping errors [12]. Thus, we maintain that adoption is the most likely cause for female-pup mismatches within our dataset.

### Population-level effects of alloparenting

Discussion of the population-level effects of an altruistic trait such as alloparenting frequently centers on the topic of Multilevel Selection Theory which explains the evolution of such traits via their advantage to the group [23], [24]. We stress that we are not approaching the connection between alloparental behavior and population viability as advocates for the support of this, or any, theory on the persistence of altruistic traits in a population. Rather, our goal is to better understand the ecological impacts associated with alloparental behavior as they apply to the fields of population and conservation biology.

Under the right circumstances, alloparenting may help maintain population size and persistence. We show that adoption in a California sea lion colony has the potential to influence long-term population growth and that these population-level benefits can be seen even when adoption is infrequent (Figure 4). A positive response of population growth to alloparental behavior is intuitive under the assumption that there are no associated reproductive costs. Support for this assumption is found in evidence that suggests the energetic costs of alloparenting may be negligible [3], [22], [25] or that the alloparent may benefit from the relationship, e.g. young females gain maternal experience leading to increased survival for future, filial offspring [2], [3], [19]. However, the costs and benefits of alloparental behavior are complex, poorly understood, and highly variable [1]–[3]. An enormous amount of effort, time, and expense would be necessary for more precise estimates of how adoption affects individual fitness in sea lions. Consequently, to incorporate a cost to adoption in our model, we were restricted to a purely hypothetical scenario. For the purpose of brevity we chose to illustrate an effect of cost using one such scenario, although we acknowledge that there are many other possible scenarios.

We predict that alloparental behavior will have a positive effect on λ when the sum of its effects on lifetime reproductive output for the alloparent and beneficiary is positive. This will occur when alloparental behavior i) provides a neutral or positive effect on the reproductive output of the alloparent or ii) provides a net increase in the reproductive output of the beneficiary that is greater than the net decrease of reproductive output incurred by the alloparent. This assumes that there is no difference between the fitness of offspring produced by the alloparent and offspring produced by the beneficiary. Our results are broadly relevant for all forms of alloparental care and across taxa.

## Supporting Information

Table S1 The number of observed alleles and expected heterozygosity (HE) for each locus.
(0.04 MB DOC)
