# Supplementary material for: First Evidence for Adoption in California Sea Lions
Source: PLoS One. 2010 Nov 8;5(11):e13873. doi: 10.1371/journal.pone.0013873 (PMC2975628; doi:10.1371/journal.pone.0013873)
Supplement: Table S1 — The number of observed alleles and expected heterozygosity (HE) for each locus. (0.04 MB DOC) [file pone.0013873.s001.doc]

**Table S1**: The number of observed alleles and expected heterozygosity (H_E_) for each locus. Calculations were conducted separately for each rookery.

|  | San Jorge | | Los Islotes | |
| --- | --- | --- | --- | --- |
| Locus | Alleles | H_E_ | Alleles | H_E_ |
| Pv09^1,2^ | 6 | 0.482 | 7 | 0.496 |
| Pv11^1,2^ | 4 | 0.488 | 5 | 0.544 |
| ZcCgDh4.7^2^ | 3 | 0.460 | 3 | 0.461 |
| ZcCgDh5.16^2^ | 9 | 0.782 | 6 | 0.693 |
| ZcCgDh1.8^2^ | 6 | 0.663 | 6 | 0.666 |
| ZcCgDh48^2^ | 4 | 0.619 | 5 | 0.536 |
| ZcCgDh5.8^2^ | 10 | 0.756 | 11 | 0.815 |
| OrrFCB24^3,4^ | 11 | 0.850 | 11 | 0.840 |
| Pvc29^4,5^ | 16 | 0.842 | 16 | 0.880 |
| ZcCgDh3.6^2^ | 6 | 0.630 | 7 | 0.551 |
| Hg6.1^2,6^ | 5 | 0.635 | 9 | 0.687 |
| Hg8.10^4,6^ | 5 | 0.541 | 7 | 0.683 |
| 13HDZ462^7^ | 4 | 0.518 | 4 | 0.634 |
| 71HDZ5A^7^ | 9 | 0.632 | 9 | 0.737 |
| Average | 7.00 | 0.6355 | 7.57 | 0.6611 |

1. Goodman SJ, 1997. Dinucleotide repeat polymorphisms at seven anonymous microsatellite loci cloned from the European harbour seal (*Phoca vitulina vitulina*). Animal Genetics 28:310-311.

2. Hernandez-Velazquez FD, Galindo-Sanchez CE, Taylor MI, De La Rosa-Velez J, Cote IM, Schramm Y, Aurioles-Gamboa D, Rico C, 2005. New polymorphic microsatellite markers for California sea lions (*Zalophus californianus*). Molecular Ecology Notes 5:140-142.

3. Buchanan FC, Maiers LD, Thue TD, De March BGE, Stewart REA, 1998. Microsatellites from the Atlantic walrus *Odobenus rosmarus rosmarus*. Molecular Ecology 7:1083-1084.

4. Wolf JBW, Tautz D, Caccone A, Steinfartz S, 2006. Development of new microsatellite loci and evaluation of loci from other pinniped species for the Galápagos sea lion (*Zalophus californianus wollebaeki*). Conservation Genetics 7:461-465.

5. Coltman DW, Bowen WD, J.M. W, 1996. PCR primers for harbor seal (*Phoca vitulina concolour*) microsatellites amplify polymorphic loci in other pinniped species. Molecular Ecology 5:161-163.

6. Allen PJ, Amos W, Pomeroy PP, Twiss SD, 1995. Microsatellite variation in grey seals, *Halichoerus grypus*: a study using DNA fingerprinting. Proceedings of the Royal Society of London Series B-Biology Sciences 252.

7. Hubinger RM, Louis EEJ, Gelatt T, Rea LD, Bickham JW, 2007. Characterization of eight microsatellite loci in Steller sea lions (*Eumetopias jubatus)*. Molecular Ecology Notes 7:1097-1099.
